# c-FLIP and CD95 signaling are essential for survival of renal cell carcinoma

**DOI:** 10.1038/s41419-019-1609-y

**Published:** 2019-05-16

**Authors:** Tobias Luebke, Lisa Schwarz, Yan Yan Beer, Sabrina Schumann, Maria Misterek, Frida Ewald Sander, Carlos Plaza-Sirvent, Ingo Schmitz

**Affiliations:** 1 Institute of Molecular and Clinical Immunology, Otto-von-Guericke University, Leipziger Straße 44, 39120 Magdeburg, Germany; 2 Systems-Oriented Immunology and Inflammation Research Group, Helmholtz Centre for Infection Research, Inhoffenstraße 7, 38124 Braunschweig, Germany

**Keywords:** Renal cell carcinoma, Apoptosis

## Abstract

Clear cell renal cell carcinoma (ccRCC) is the most-prominent tumor type of kidney cancers. Resistance of renal cell carcinoma (RCC) against tumor therapy is often owing to apoptosis resistance, e.g., by overexpression of anti-apoptotic proteins. However, little is known about the role of the apoptosis inhibitor c-FLIP and its potential impact on death receptor-induced apoptosis in ccRCC cells. In this study, we demonstrate that c-FLIP is crucial for resistance against CD95L-induced apoptosis in four ccRCC cell lines. Strikingly, downregulation of c-FLIP expression by short hairpin RNA (shRNA)interference led to spontaneous caspase activation and apoptotic cell death. Of note, knockdown of all c-FLIP splice variants was required to induce apoptosis. Stimulation of ccRCC cells with CD95L induced NF-κB and MAP kinase survival pathways as revealed by phosphorylation of RelA/p65 and Erk1/2. Interestingly, CD95L surface expression was high in all cell lines analyzed, and CD95 but not TNF-R1 clustered at cell contact sites. Downstream of CD95, inhibition of the NF-κB pathway led to spontaneous cell death. Surprisingly, knockdown experiments revealed that c-FLIP inhibits NF-κB activation in the context of CD95 signaling. Thus, c-FLIP inhibits apoptosis and dampens NF-κB downstream of CD95 but allows NF-κB activation to a level sufficient for ccRCC cell survival. In summary, we demonstrate a complex CD95-FLIP-NF-κB-signaling circuit, in which CD95-CD95L interactions mediate a paracrine survival signal in ccRCC cells with c-FLIP and NF-κB both being required for inhibiting cell death and ensuring survival. Our findings might lead to novel therapeutic approaches of RCC by circumventing apoptosis resistance.

## Introduction

Renal cell carcinoma (RCC) accounts for 3.8% of malignancies of the adult and over 90% of kidney tumors^1^. RCC is a cancer of the proximal renal tubular epithelium and the collecting tubular epithelium and comprises a variety of malignancies that differ in histology and on the molecular level^1–3^. One particular type of RCC, the clear cell RCC (ccRCC), often exhibits mutations in the von Hippel-Lindau (VHL) tumor suppressor gene and in PBRM1, a component of the PBAF chromatin-remodeling complex^4^. Another main characteristic of RCC is the resistance toward apoptosis-inducing chemotherapeutic approaches^5^. Therefore, previous therapies were based on cytokines such as interleukin-2 or interferon-α to boost immune responses^1,6^. More recently, therapy switched to molecular approaches targeting multiple tyrosine kinases or the mammalian target of rapamycin^1,6^. Although the median survival time was increased by the molecular therapies, kidney cancer still accounted for 143,000 deaths in 2012^2,6^. Therefore, additional molecular therapies are urgently needed for the treatment of RCC.

Resistance to apoptosis is a hallmark of cancer cells and can lead to tumor formation^7^. One important molecular pathway, called extrinsic apoptosis, is initiated by death ligands like CD95L-, TNFα-, and TRAIL-triggering activation of their respective receptors^8^. Upon ligand binding, the protein Fas-associated protein with death domain (FADD) binds to CD95 and acts as recruitment platform for death effector domain (DED)-containing proteins such as caspase-8 (formerly known as FLICE) and caspase-10 forming the death inducing signaling complex (DISC)^8,9^. When two caspase molecules come into close proximity, they undergo self-cleavage into an active form by removing the N-terminal DED domains^10,11^. The activated caspase is then released from the DISC activating downstream effector caspases, leading to apoptotic death of the cell^12,13^.

The apoptotic machinery is controlled by various anti-apoptotic proteins, whose expression is tightly regulated. For instance, cellular FLICE-like inhibitory proteins (c-FLIP) can inhibit apoptosis at the DISC^14^. Three different c-FLIP splice variants (c-FLIP_L_, c-FLIP_S_, and c-FLIP_R_) have been identified so far that are expressed on the protein level^14–17^. All three c-FLIP proteins have, like caspase-8 and caspase-10, two DEDs, which are crucial for DISC recruitment, inhibition of caspase activation and apoptosis execution^14,18^. Although c-FLIP is regarded primarily as an anti-apoptotic protein, the three isoforms can have different functions. Although only anti-apoptotic functions have been described for c-FLIP_S_ and c-FLIP_R_, c-FLIP_L_ was also shown to have pro-apoptotic features, which depend on the presence of the C-terminal caspase-like-domain that itself is catalytically inactive but allows activation of caspase-8 when forming a heterodimer^19,20^. The different properties of c-FLIP_L_ in apoptosis regulation are determined by the expression levels of all c-FLIP splice variants as well as the stimulation strength of the death receptors^21^. In addition, when c-FLIP_L_ is cleaved, a p43-FLIP fragment is generated, which was shown to interact with RIP1 and TRAF2 and thereby activating the nuclear factor “κ-light-chain-enhancer“ of activated B cells (NF-κB)^22–24^. On the other hand, c-FLIP has also been reported to inhibit death receptor-mediated NF-κB activation^25–27^.

Anti-apoptotic proteins are known to be upregulated in several tumor types. For instance, inhibition of the extrinsic pathway allows cells having the potential for tumor formation to evade immune surveillance mechanisms, which recognize mutated and potentially defective cells^7^. In this regard, upregulation of c-FLIP was for example shown in breast cancer^28^, melanoma^17,29^, and Hodgkin’s lymphoma^30,31^. Furthermore, expression of c-FLIP can be a resistance factor in colon cancer^32^, non-small cell lung cancer^33^, and urothelial carcinoma^34^. However, a contribution of c-FLIP splice variants in promoting apoptosis resistance in RCC has not been addressed in previous studies.

To uncover apoptosis resistance mechanisms in RCC, we characterized four different clear cell RCC (termed clearCa) cell lines, which are protected against TRAIL-mediated apoptosis^35^. Surprisingly, we found that clearCa cell lines critically depended on c-FLIP expression. In this regard, our study revealed a crucial function of c-FLIP and the CD95 system for survival of clearCa cells. Targeting this signaling axis may provide novel therapy options for ccRCC.

## Results

### c-FLIP mediates resistance against CD95L-induced apoptosis

**Fig. 1:**
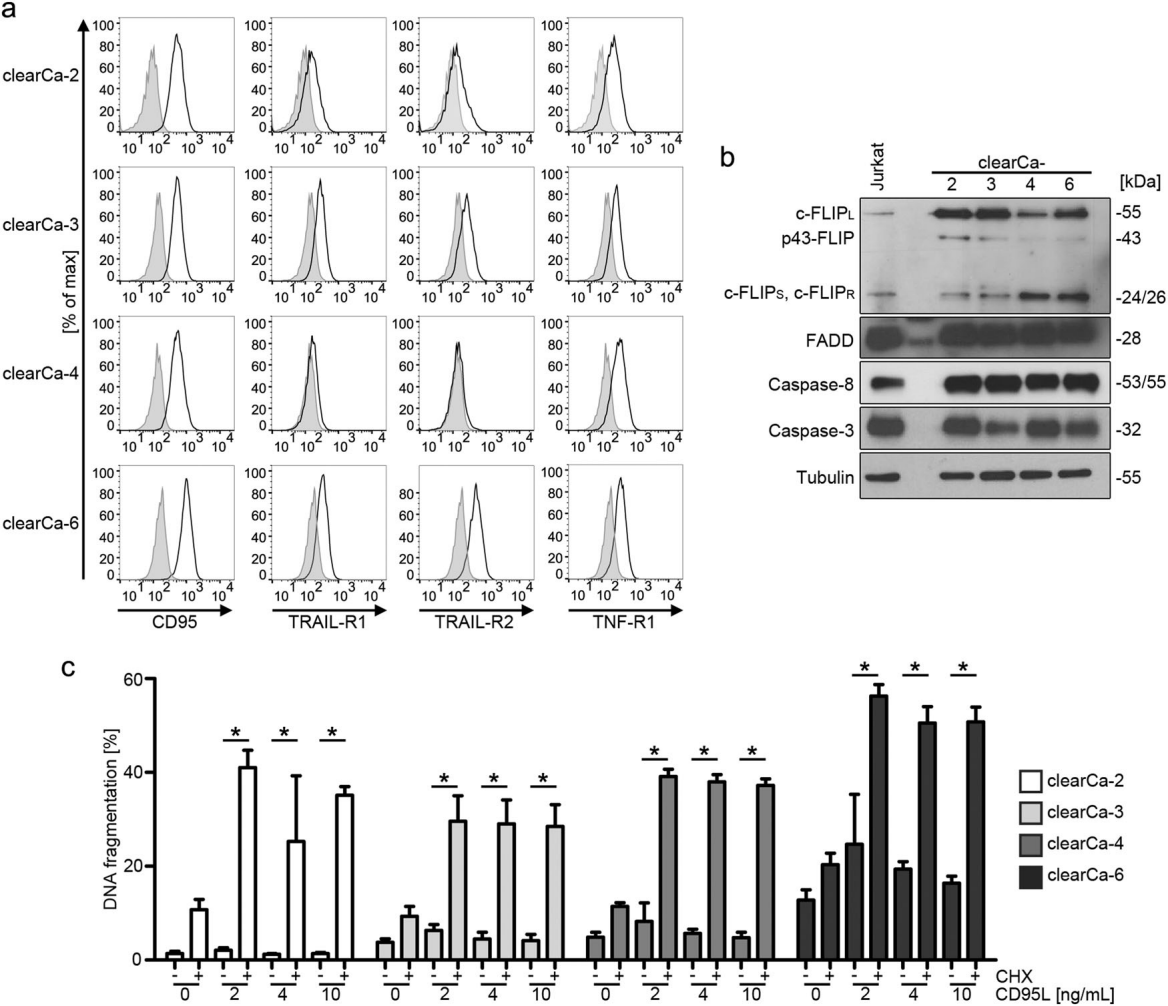
Cycloheximide sensitizes clearCa cells towards CD95L-induced apoptosis. **a** Surface expression of death receptors CD95, TRAIL-R1, TRAIL-R2, or TNF-R1 (black line) on clearCa-2, -3, -4, and -6 cells was detected by flow cytometry with specific antibodies. Unstained samples are shown in gray. **b** Expression levels of the DISC proteins c-FLIP, FADD, and caspase-8 as well as caspase-3 in clearCa-2, -3, -4, and -6 cells were analyzed via immunoblotting. Tubulin served as loading control. **c** Analysis of DNA fragmentation after stimulation of clearCa cells with 0, 2, 4, or 10 ng/mL CD95L in the presence or absence of 10 µg/mL CHX for 16 h. Bars display the mean of at least three experiments, error bars represent SD. Statistical significances were calculated by one-tailed Mann–Whitney *U* test; * *p* *≤* 0.05

**Fig. 2:**
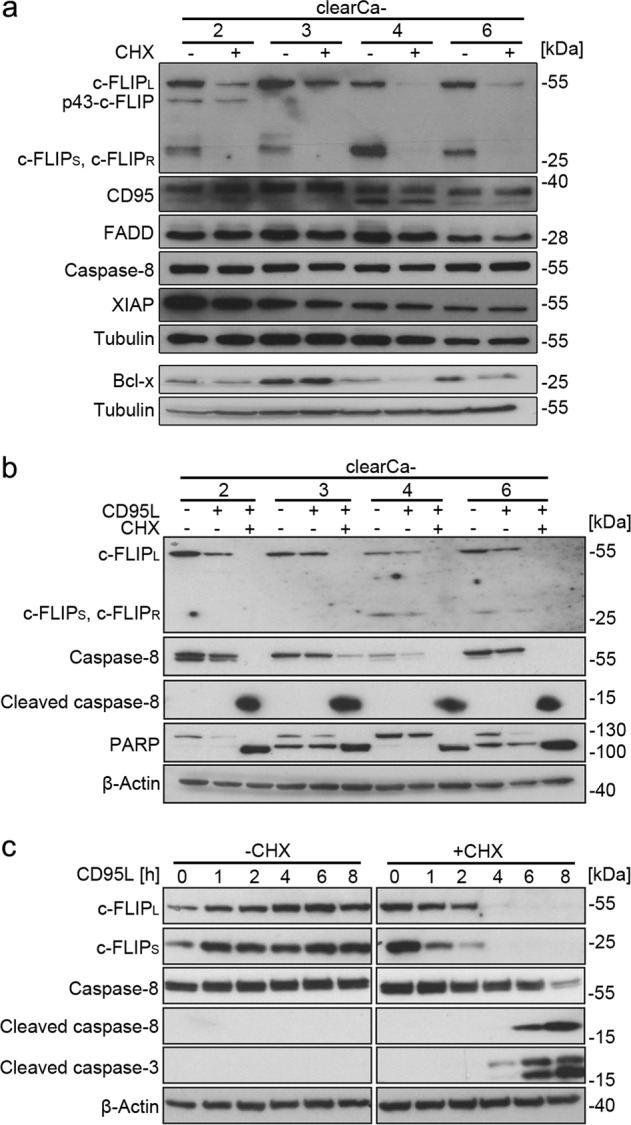
Identification of c-FLIP as the cycloheximide-sensitive resistance factor in clearCa cells. **a** Analysis of the influence of cycloheximide on the expression levels of anti-apoptotic proteins. Cells were treated with 10 µg/mL CHX for 8 h and analyzed by immunoblotting for expression of the indicated proteins. Tubulin was used as loading control. **b** Immunoblot analysis of c-FLIP expression, caspase-8 activation and PARP cleavage upon CD95L-induced apoptosis after stimulation with 10 ng/mL CD95L in the presence or absence of 10 µg/mL CHX for 8 h. β-Actin was used as loading control. **c** clearCa-4 cells were treated with 10 ng/mL CD95L in the presence or absence of 10 µg/mL CHX for up to 8 h. Activation of caspase-8 and caspase-3 as well as c-FLIP expression was analyzed by immunoblot analysis. β-Actin was used as loading control

We characterized the expression of DISC-related proteins in four different ccRCC cell lines (clearCa-2, -3, -4, and -6). Analysis of death receptor expression by surface staining revealed high levels of CD95 and intermediate TNF-R1 expression in all cell lines. TRAIL-R1 and TRAIL-R2 expression was detectable in clearCa-2, -3, and -6, whereas it was absent in clearCa-4 (Fig. 1a). The intracellular DISC components FADD and caspase-8 were expressed equally between the four clearCa cell lines (Fig. 1b). The apoptosis inhibitor c-FLIP was expressed by all four cell lines analyzed, with c-FLIP_L_ expression being higher than expression of the short splice variants c-FLIP_S_ and c-FLIP_R_ in clearCa-2 and -3 cell lines (Fig. 1b). Expression of the long and the short c-FLIP splice variants were comparable in clearCa-4 and clearCa-6. Genomic analysis of the 3’ splicing site of intron 6 in the *CFLAR* gene revealed heterogeneous expression of the short splice variants c-FLIP_S_ and c-FLIP_R_. clearCa-3 and -6 were heterozygous for c-FLIP_S_ and c-FLIP_R_, clearCa-2 was homozygous for c-FLIP_R_ and clearCa-4 was homozygous for c-FLIP_S_ (data not shown). We then tested all cell lines for CD95L-induced apoptosis. Cells were stimulated with 2, 4, or 10 ng/mL recombinant CD95L for 16 h in the presence or absence of the protein translation inhibitor cycloheximide (CHX) and cell death rates were measured by analyzing the sub-G1 DNA peak of propidium iodide stained cells. At the concentrations tested, all four cell lines were resistant against stimulation with CD95L alone, but were significantly sensitized by addition of CHX (Fig. 1c). Treatment of cells with CD95L alone was not sufficient for caspase-8 activation. Upon treatment of clearCa cells with CHX, we detected distinct downregulation of the short-lived c-FLIP proteins (Fig. 2a). In contrast, expression of XIAP was only marginally affected and Bcl-x_L_ was downregulated in only some of the cell lines analyzed (Fig. 2a). As c-FLIP blocks CD95L-induced apoptosis at the level of the DISC, it is the most likely candidate for promoting CD95L-induced apoptosis resistance in clearCa cell lines. In line, combined stimulation of clearCa cells with CD95L and CHX revealed loss of c-FLIP expression, activation of caspase-8 and PARP cleavage (Fig. 2b). Moreover, downregulation of c-FLIP proteins upon CHX treatment preceded activation of caspase-8 and caspase-3 (Fig. 2c).

### c-FLIP is essential for survival of clearCa cell lines

**Fig. 3:**
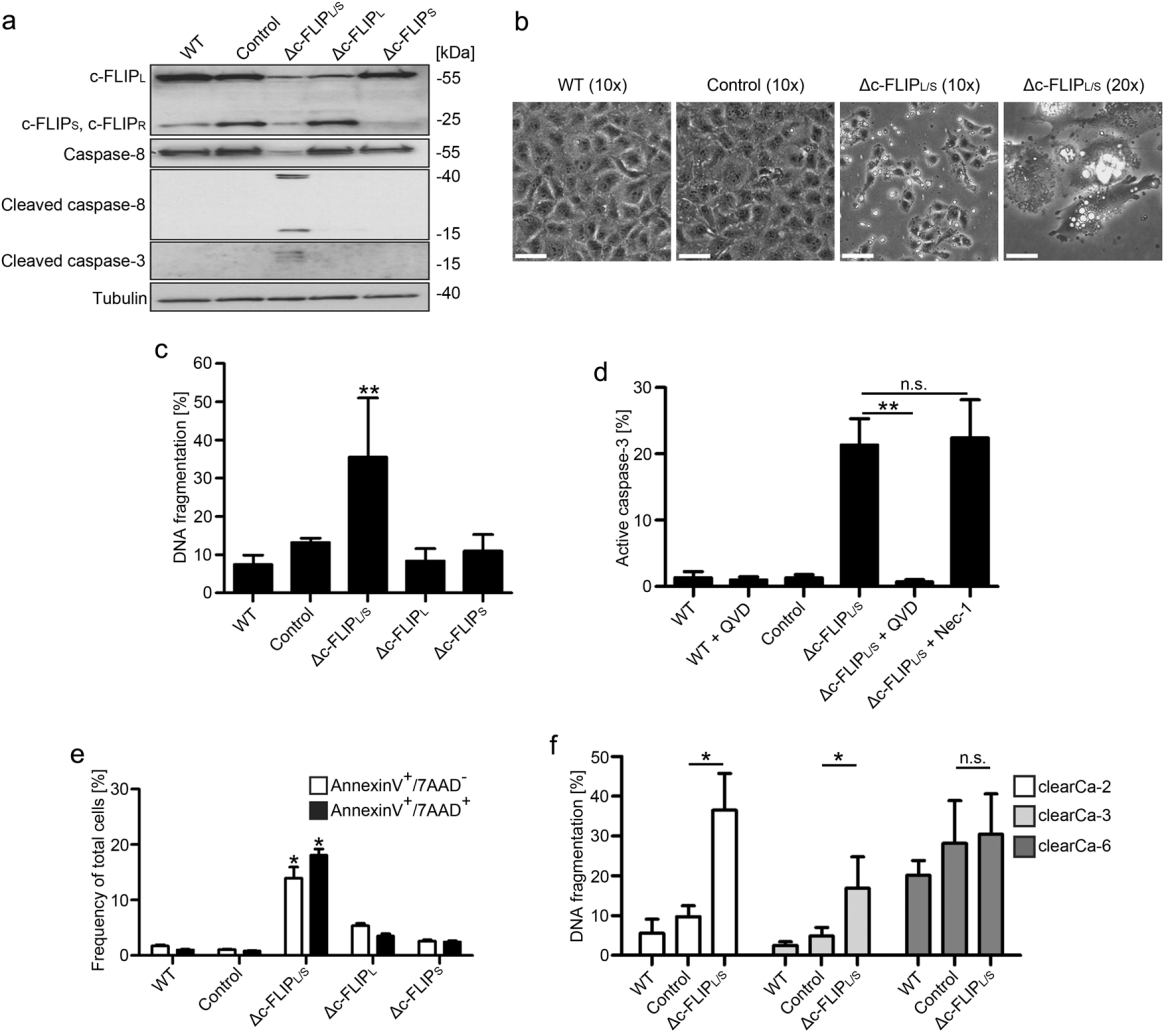
Knockdown of c-FLIP induces spontaneous apoptosis in clearCa cells. **a** clearCa-4 cells were transduced with lentiviral constructs encoding shRNAs for all c-FLIP isoforms (Δc-FLIP_L/S_) or for specific splice variants (Δc-FLIP_L_ and Δc-FLIP_S_, respectively). A scrambled shRNA construct was used as control. Efficiency of c-FLIP knockdown and caspase activation was verified by immunoblot analysis three days after lentiviral transduction. **b** Microscopic pictures were taken on day 3 after lentiviral transduction of scrambled shRNA (control) or shRNA for all c-FLIP isoforms (Δc-FLIP_L/S_). White bar represents 100 µm (× 10 magnification) or 50 µm (× 20 magnification). **c** clearCa-4 cells were transduced as in **a**. Cell death was assessed on day 3 post transduction by analysis of DNA fragmentation. **d** clearCa-4 cells were transduced with a lentiviral construct encoding shRNA for knockdown of all c-FLIP isoforms (Δc-FLIP_L/S_). QVD and Nec-1 was added to block caspase-mediated apoptosis and Ripk1-mediated necroptosis, respectively. Subsequently, cells stained for intracellular active caspase-3. **e** clearCa-4 cells were transduced as in **a** and cell death was analyzed by AnnexinV/7AAD staining. **f** Analysis of cell death in clearCa-2, -3, and -6 cells after lentiviral knockdown of all c-FLIP isoforms on day 3 after transduction. A scrambled shRNA construct was used as control. Cell death was analyzed by quantifying DNA fragmentation via flow cytometry. Bars display the mean of at least three experiments, error bars represent SD. Statistical significances were calculated by one-tailed Mann–Whitney *U* test in respect to Control sample; * *p* *≤* 0.05

To analyze the impact of c-FLIP on mediating apoptosis resistance, clearCa-4 was chosen for further analysis. clearCa-4 cells expressed comparable amounts of c-FLIP_L_ and c-FLIP_S_ and were the only cell line in the panel that lacked c-FLIP_R_ expression against which no short hairpin RNA (shRNA) exists. We employed a shRNA approach for the downregulation of particular c-FLIP splice variants to understand potential functional differences between c-FLIP_L_ and c-FLIP_S_ in apoptosis resistance. clearCa-4 cells were treated with lentiviral constructs, targeting either c-FLIP_L_ (Δc-FLIP_L_), c-FLIP_S_ (Δc-FLIP_S_), or all splice variants c-FLIP_L_, c-FLIP_S_, and c-FLIP_R_ (Δc-FLIP_L/S_) or containing a scrambled shRNA. All three c-FLIP-targeting constructs were potent in downregulating their respective targeted isoform, as can be seen by the diminished expression of c-FLIP_L_, c-FLIP_S_, and c-FLIP_L_ plus c-FLIP_S_, respectively (Fig. 3a). Downregulation of c-FLIP_L_ or c-FLIP_S_ alone showed no effect on caspase activation and survival of clearCa-4 cells even at day 5 post transduction (data not shown). Surprisingly, Δc-FLIP_L/S_ cells showed spontaneous caspase-8 and caspase-3 activation at day 3 post shRNA delivery (Fig. 3a). No differences in Δc-FLIP_L_ or Δc-FLIP_S_ cells were detectable, suggesting that apoptosis resistance is equally mediated by both splice variants. Microscopic analysis revealed high rates of dead cells with apoptotic morphological features such as membrane blebbing for Δc-FLIP_L/S_ treated cells (Fig. 3b). In addition, we analyzed DNA fragmentation after 3 days of treatment, which was significantly elevated in Δc-FLIP_L/S_ cells but not single knockdown cells (Fig. 3c), supporting the notion that the presence of one c-FLIP splice variant is sufficient to prevent spontaneous cell death. To proof an apoptotic mode of action, Δc-FLIP_L/S_ cells were additionally treated with the caspase inhibitor QVD-OPh (QVD) or the necroptosis inhibitor Necrostatin-1 (Nec-1) and analyzed 3 days later by flow cytometry. Treatment of cells with QVD completely blocked intracellular caspase-3 activation, whereas Nec-1 had no effect on caspase-3 activation (Fig. 3d). We also studied loss of plasma membrane asymmetry by staining of phosphatidylserine with fluorescent AnnexinV, followed by flow cytometric analysis, in wild type, scrambled shRNA-treated, Δc-FLIP_L_, Δc-FLIP_S_, and Δc-FLIP_L/S_ cells. Elevated numbers of AnnexinV-positive cells were detectable in Δc-FLIP_L/S_ compared with wild-type cells (Fig. 3e). To follow the course of apoptosis, complete disintegration of the cell membrane was shown by concurrent DNA staining with 7AAD. As for AnnexinV, 7AAD-positive cells were detectable in Δc-FLIP_L/S_ cells only (Fig. 3e). Complete loss of c-FLIP also induced spontaneous apoptosis of clearCa-2 and clearCa-3 cells, showing a dependency on c-FLIP of these cell lines as well (Fig. 3f). Taken together, loss of c-FLIP leads to spontaneous apoptosis in clearCa cell lines, whereas expression of one isoform is sufficient for cell viability. Thus, splice variant-independent c-FLIP expression seems to be necessary for clearCa survival in three of four cell lines (clearCa-2, -3, and -4).

### Re-expression of c-FLIP_L_ restores cell viability of Δc-FLIP_L/S_ cells to wild-type levels

**Fig. 4:**
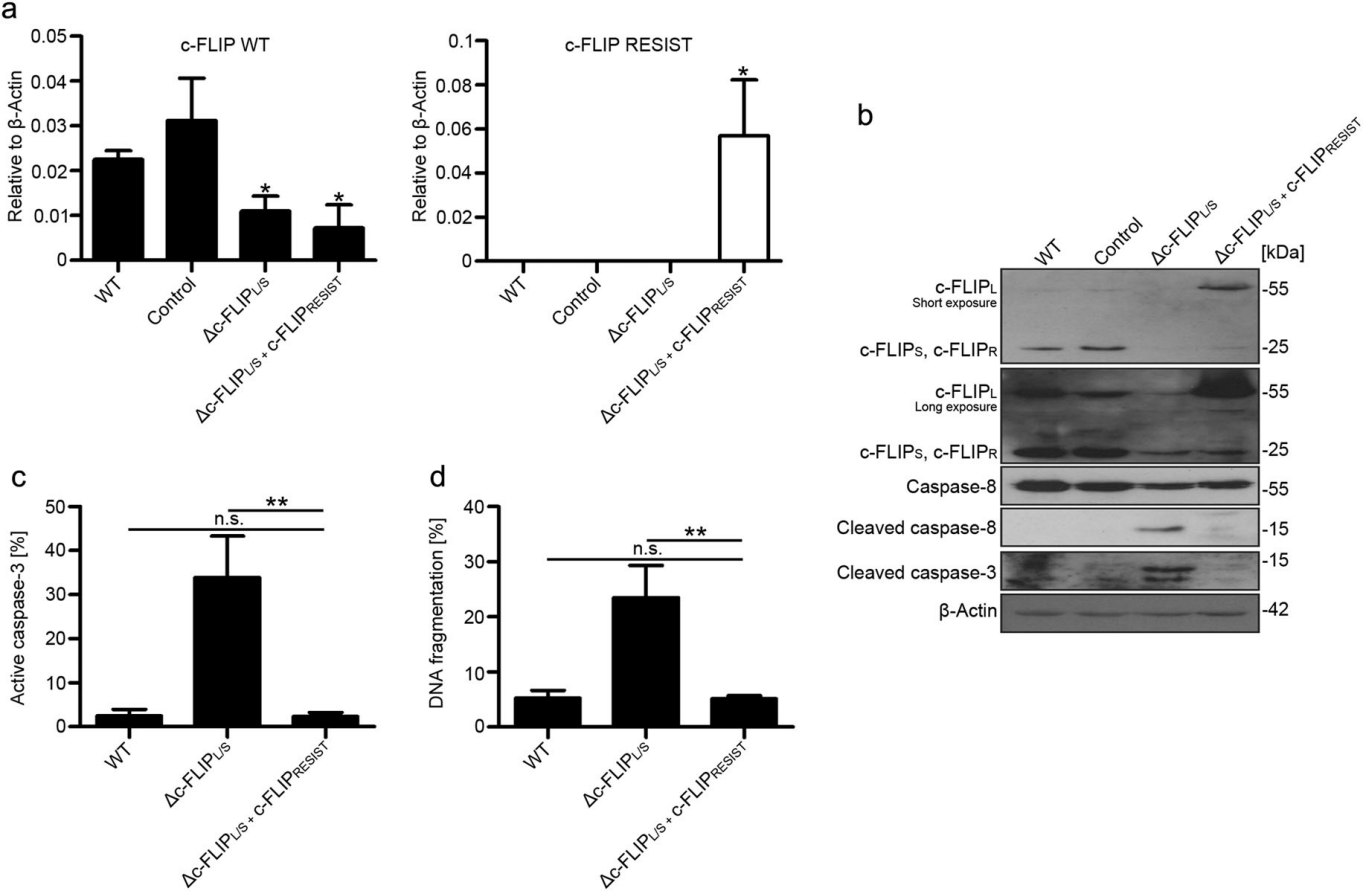
Re-expression of c-FLIP_L_ protects clearCa-4 cells from c-FLIP knockdown-induced apoptosis. **a** Cells were transduced with either a control, a c-FLIP_L/S_-targeting, or a c-FLIP_L/S_ targeting lentiviral construct that simultaneously expresses a shRNA-resistant version of c-FLIP_L_ (c-FLIP_RESIST_). Transduced cells were analyzed 3 days after lentiviral shRNA delivery. Expression of c-FLIP mRNA was analyzed with specific primers for wild type or mutated c-FLIP mRNA. Bars display the mean of three experiments, error bars represent SD; *p *≤* 0.05. **b** Cells were transduced as in **a**. Immunoblot analysis of c-FLIP expression levels and activation of caspase-3 and caspase-8. β-Actin was used as loading control. **c** Cells were transduced as in **a** and subsequently analyzed for the expression of intracellular active caspase-3 by flow cytometry. **b** Cell were transduced as in **a** and cell death was analyzed via flow cytometrical quantification of DNA fragmentation. Bars display the mean of three experiments, error bars represent SD. Statistical significances were calculated by one-tailed Mann–Whitney *U* test; n.s. = not significant, **p *≤* 0.01

To exclude any effects of the lentiviral treatment on cell survival or side effects of the used shRNA on other essential survival factors, we generated a construct to re-express a shRNA-resistant form of c-FLIP_L_ while blocking the expression of wild-type c-FLIP_L_ and c-FLIP_S_. For re-expression, we inserted silent mutations in the shRNA target sequence of the c-FLIP_L_ gene and exchanged the resistance cassette against the mutated c-FLIP_L_ cDNA (Δc-FLIP_L/S_ + c-FLIP_RESIST_). The capability of the construct to downregulate wild-type c-FLIP mRNA and simultaneous re-expression of c-FLIP_RESIST_ mRNA was confirmed via quantitative real-time PCR with primers specific for endogenous and shRNA-resistant c-FLIP, respectively. Compared with wild-type cells, c-FLIP mRNA was significantly reduced in Δc-FLIP_L/S_ and Δc-FLIP_L/S_ + c-FLIP_RESIST_ cells. Amplification of c-FLIP_RESIST_ mRNA was only detectable in cells transduced with the re-expression construct (Fig. 4a), showing that the inserted mutations were sufficient to prevent mRNA degradation by c-FLIP_L/S_-targeting shRNA. On the protein level, expression of c-FLIP_S_ was downregulated in Δc-FLIP_L/S_ and Δc-FLIP_L/S_ + c-FLIP_RESIST_ cells, but levels of c-FLIP_L_ were considerably higher in Δc-FLIP_L/S_ + c-FLIP_RESIST_ cells compared with wild-type cells, confirming the re-expression of c-FLIP_RESIST_ while preventing expression of endogenous c-FLIP (Fig. 4b). To proof functionality of the re-expressed c-FLIP_RESIST_, we compared caspase activation and cell death rates of Δc-FLIP_L/S_ and Δc-FLIP_L/S_ + c-FLIP_RESIST_ cells. Caspase-8 was not activated in Δc-FLIP_L/S_ + c-FLIP_RESIST_ cells (Fig. 4b). In addition, intracellular active caspase-3 was significantly reduced upon re-expression of c-FLIP_RESIST_ in comparison with Δc-FLIP_L/S_ cells (Fig. 4c). Vitality of the cells was measured via DNA fragmentation, which revealed that re-expression of c-FLIP_RESIST_ was sufficient to restore cell viability in Δc-FLIP_L/S_ + c-FLIP_RESIST_ cells to wild-type levels (Fig. 4d). Taken together, c-FLIP re-expression rescued the Δc-FLIP_L/S_ phenotype of spontaneous cell death.

### The CD95 receptor system is activating the NF-κB and ERK pathways and crucial for cell survival

**Fig. 5:**
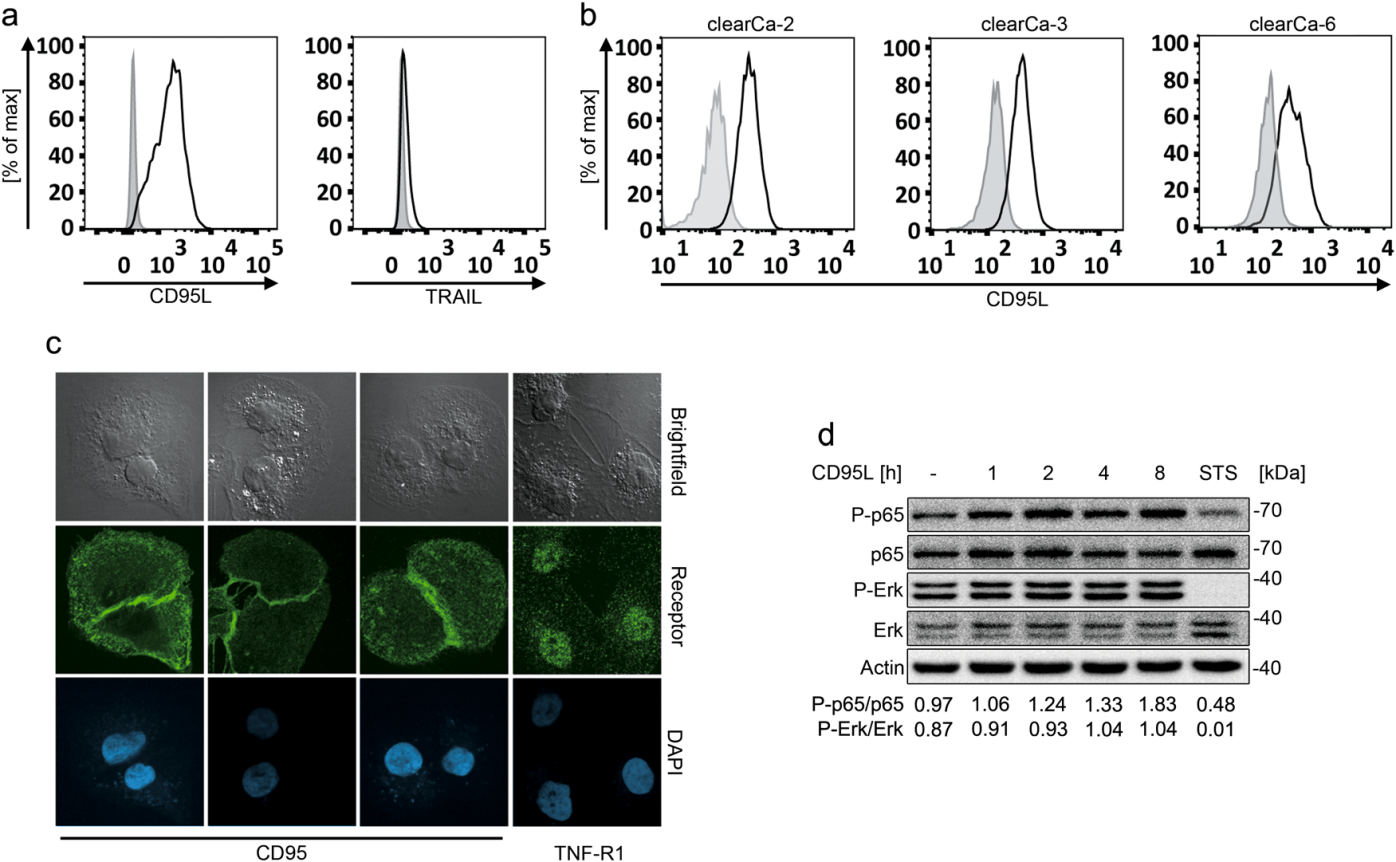
CD95-mediated signaling is essential for clearCa cell survival. **a** clearCa-4 cells were stained with antibodies against CD95L or TRAIL (black line) and analyzed by flow cytometry. Unstained samples are shown in grey. **b** Surface expression of CD95L (black line) was analyzed by flow cytometry on clearCa-2, clearCa-3, and clearCa-6 cells. Unstained samples are shown in grey. **c** CD95 and TNF-R1, respectively, were stained on clearCa-4 and analyzed by fluorescence microscopy. Receptors are depicted in green. Nuclei were stained with DAPI (blue). For each condition, 50 pictures with cell–cell contacts were analyzed; representative samples are shown. **d** clearCa-4 cells were stimulated with 10 ng/mL CD95L for up to 8 h. NF-κB and MAP kinase activation was assessed by detection of phospho-p65 and phospho-Erk by immunoblot analysis. Tubulin, total p65, and total Erk were used as loading control

Next to apoptosis regulation, CD95 and c-FLIP were also shown to activate NF-κB and ERK^22–24,36–38^ and CD95 expression is high in all clearCa cell lines (Fig. 1a). Therefore, we wanted to know whether clearCa cells utilized the CD95 pathway for induction of the NF-κB and MAP kinase survival pathways. First, we analyzed CD95L surface expression by flow cytometry. ClearCa-4 cells expressed high levels of CD95L, but not TRAIL (Fig. 5a). Similarly, the expression level of CD95L was high in clearCa-2, -3, and -6 cells (Fig. 5b). In addition, CD95 surface expression pattern was analyzed via fluorescence microscopy. Interestingly, CD95 is clustered at cell–cell contacts as can be seen by the staining intensity at the contact areas of two cells (Fig. 5c). As clusters of CD95 may lead to DISC formation and subsequent c-FLIP-dependent activation of survival pathways, we analyzed phosphorylation of the RelA/p65 NF-κB subunit as well as the MAP kinases Erk1/2. Phosphorylation of RelA/p65 was already detectable in untreated cells (Fig. 5d). Stimulation with 10 ng/mL CD95L increased RelA/p65 phosphorylation, indicating NF-κB activation (Fig. 5d). Similarly, phosphorylation of the MAP kinase Erk1/2 increased upon CD95L stimulation of clearCa-4 cells (Fig. 5d).

**Fig. 6:**
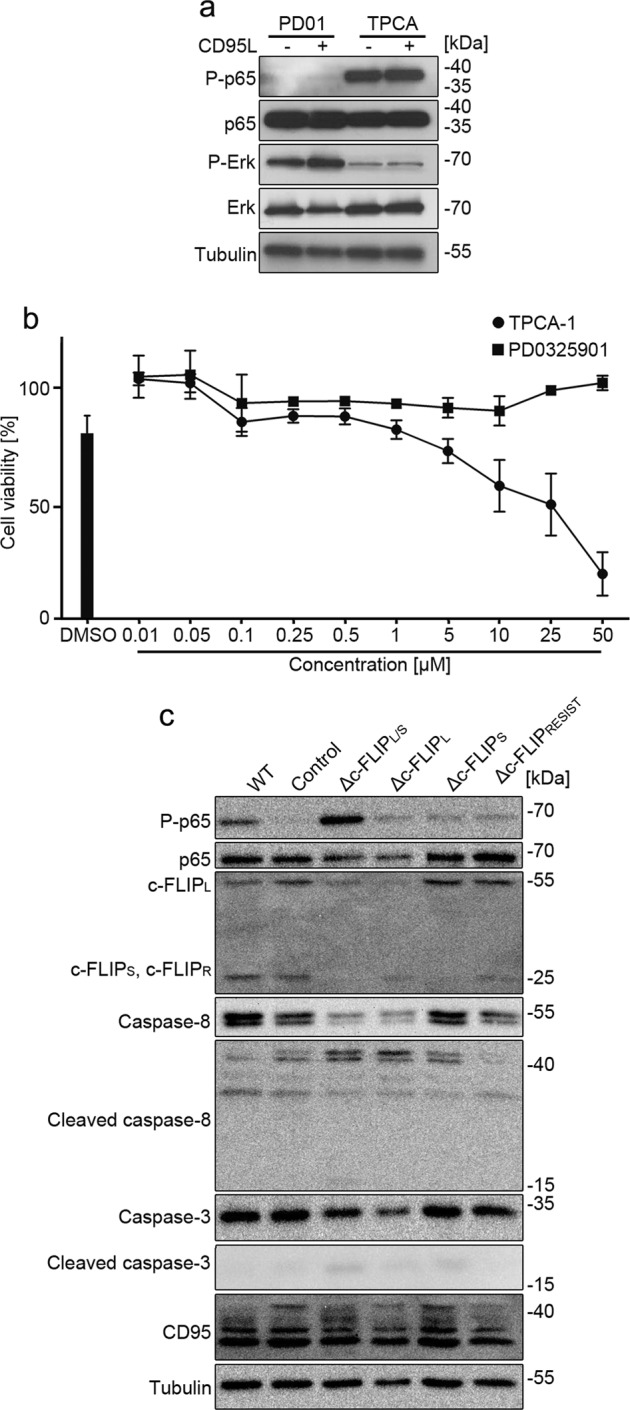
CD95L-induced NF-κB is essential for clearCa cell survival and c-FLIP inhibits NF-κB in the context of CD95 signaling. **a** ClearCa-4 cells were treated with 50 µm TPCA-1 or 10 µm PD0325901 in presence or absence of 10 ng/ml CD95L for 8 h and analyzed with phospho-p65 and p-Erk antibodies. Total p65, total Erk and tubulin were used as loading controls. **b** ClearCa-4 cells were treated with the indicated concentrations of the NF-κB inhibitor TPCA-1 and the MEK1 inhibitor PD0325901, respectively, for 24 h. Subsequently, cell viability was determined by resazurin assay. **c** clearCa-4 cells were transduced with lentiviral constructs encoding shRNAs for all c-FLIP isoforms (Δc-FLIP_L/S_) or for specific splice variants (Δc-FLIP_L_ and Δc-FLIP_S_, respectively) or a c-FLIP_L/S_ targeting lentiviral construct that simultaneously expresses a shRNA-resistant version of c-FLIP_L_ (c-FLIP_RESIST_). A scrambled shRNA construct was used as control. On day 3 post transduction, cells were lysed and analyzed by immunoblotting for the indicated proteins. Tubulin was used as a loading control

To see if the activation of NF-κB or Erk MAP kinases is necessary for cell survival, we treated ClearCa-4 cells with the IKK-β inhibitor TPCA-1, and the MEK1 inhibitor PD0325901. Both compounds inhibited the activation of the respective target (Fig. 6a). Subsequently, cell viability was determined by resazurin assay. Importantly, TPCA-1 but not PD0325901 reduced viability of clearCa-4 cells showing that activation of IKK-β but not Erk1/2 is required for cell survival (Fig. 6b).

c-FLIP has been shown not only to induce NF-κB via interaction with the IKK complex, but also to inhibit death receptor-induced NF-κB activity^25–27^. In order to investigate the function of c-FLIP in ccRCC cells, we performed knockdown experiments and analyzed the phosphorylation status of RelA/p65. Although lentiviral transduction of a scrambled shRNA into clearCa-4 cells reduced RelA/p65 phosphorylation, Δc-FLIP_L/S_ cells clearly had higher levels of phosphorylated RelA/p65 (Fig. 6c). Despite high NF-κB activity, Δc-FLIP_L/S_ cells also had high levels of cleaved caspase-8 and cleaved caspase-3. Δc-FLIP_L_, Δc-FLIP_S_, and Δc-FLIP_L/S_ + c-FLIP_RESIST_ cells exhibited an intermediate phenotype (Fig. 6c). We conclude that c-FLIP is crucial to prevent apoptosis in ccRCC cells, but additionally dampens NF-κB activity.

**Fig. 7:**
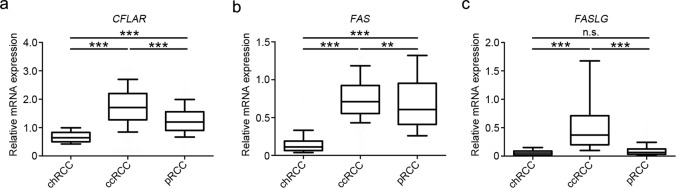
*CFLAR*, *FAS*, and *FASLG* are differently expressed in renal cell carcinomas. **a** Relative expression of *CFLAR* determined by RNASeq V2 of clear cell renal cell carcinoma (ccRCC, *n* = 1003), kidney chromophobe (chRCC, *n* = 132) and kidney renal papillary cell carcinoma (pRCC, *n* = 291). **b** Relative expression of *FAS* determined by RNASeq V2 of clear cell renal cell carcinoma (ccRCC, *n* = 534), kidney chromophobe (chRCC, *n* = 66) and kidney renal papillary cell carcinoma (pRCC, *n* = 291). **c** Relative expression of *FASLG* determined by RNASeq V2 of clear cell renal cell carcinoma (ccRCC, *n* = 534), kidney chromophobe (chRCC, *n* = 66) and kidney renal papillary cell carcinoma (pRCC, *n* = 291). Box plots with whiskers (10–90 percentiles) display expression of the respective genes in chRCC, ccRCC and pRCC. Kruskal–Wallis one-way ANOVA tests were used for statistical analysis; **p < 0.01, ***p < 0.001, n.s. = not significant

Finally, we wanted to know whether CD95L, CD95, and c-FLIP might be of general importance in RCC. In order to approach this question, we analyzed the expression of *CFLAR* (the gene encoding c-FLIP), *FAS* (the gene encoding CD95), and *FASLG* (the gene encoding CD95L) in RCC using the public data base cBioPortal^39,40^. We included data sets for ccRCC^41^, chromophobe RCC^42^, and papilliary RCC (TCGA, provisional). All three genes, *CFLAR*, *FAS*, and *FASLG*, were significantly higher expressed in ccRCC compared with the other two renal cancer types (Fig. 7). Thus, targeting the CD95 pathway or c-FLIP might be a novel option for the treatment of ccRCC.

## Discussion

To avoid cell death, tumors have established a broad variety of resistance mechanisms by downregulation of pro-apoptotic and upregulation of anti-apoptotic proteins including c-FLIP^43,44^. Accordingly, c-FLIP was shown to mediate resistance against death receptor-induced apoptosis in various tumor types^17,28,29,34,45^. In the current study, we focused on the role of c-FLIP-mediated CD95L resistance in RCC.

Here, we showed that ccRCC cell lines were resistant against CD95L-induced apoptosis in the presence of c-FLIP, but were sensitized upon inhibition of protein translation and subsequent reduction of c-FLIP. Accordingly, we detected activation of caspase-8 and caspase-3, loss of cell membrane integrity and DNA fragmentation. Surprisingly, shRNA-mediated downregulation of all c-FLIP splice variants was sufficient to induce spontaneous apoptosis in all ccRCC cell lines tested, without further death receptor stimulation. We confirmed that this effect was owing to the absence of c-FLIP only, as re-expression of mutated c-FLIP_L_ (c-FLIP_RESIST_) was sufficient to restore cell viability. Staining of death receptors revealed high levels of CD95 and CD95L on the surface of clearCa cells. Moreover, analysis of public data on the expression levels of c-FLIP (*CFLAR*), CD95 (*FAS/TNFRSF6*), and CD95L (*FASLG*) revealed significantly higher expression of these three genes in ccRCC compared with other renal cancers. We found that CD95, but not TNF-R1 formed clusters at cell–cell contact sites. In addition, stimulation with CD95L increased phosphorylation of RelA/p65 and Erk1/2. Although pharmacological inhibition of Erk1/2 activation had no effect, inhibition of the NF-κB pathway induced cell death in clearCa cells similar to knockdown of c-FLIP and inhibition of CD95L.

The finding that knockdown of c-FLIP_S/L_ alone resulted in apoptotic death of ccRCC cells was surprising as other studies reported sensitization, but not spontaneous cell death, by inhibition of c-FLIP expression. For instance, knockdown of c-FLIP resulted in higher death rates upon stimulation with death ligands in breast cancer cells^28^, melanoma cells^29^, non-small cell lung carcinoma cells^33^, and urothelial carcinoma cells^34^. To our knowledge, a single report shows that loss of c-FLIP leads to TRAIL-R2-dependent spontaneous caspase-8 activation and subsequent apoptosis in MCF-7 cells^46^, which lack caspase-3^47^, a main effector caspase in apoptosis. However, the findings by Day et al. in the MCF-7 cell line are clearly different from our observations, as they claim a ligand-independent activation of caspases upon c-FLIP knockdown. In contrast, we show that ccRCC cells express high amounts of CD95L, leading to CD95 clusters at cell–cell contact sites. Furthermore, knockdown of c-FLIP_L_ but not c-FLIP_S_ was required for spontaneous cell death of MCF-7 cells^46^. For apoptosis induction in clearCa cells, complete loss of c-FLIP was necessary. Thus, c-FLIP has a special role for survival of ccRCC cells.

In this regard, we found that CD95L stimulation of clearCa cells resulted in the activation of the NF-κB and MAP kinase survival pathways. Pharmacological analyses demonstrated that clearCa cells depend on NF-κB signaling. Our observations are consistent with a recent report that showed inhibition of proliferation and growth in soft agar of ccRCC cells upon treatment with a different IKK inhibitor than the one used in our study^48^. Interestingly, c-FLIP has been shown to activate NF-κB^22–24,36^, but it is also a NF-κB target gene itself^49^. This could suggest that activation of NF-κB via the CD95L–CD95-c-FLIP axis might engage a positive feedback loop to ensure protection from death receptor-mediated apoptosis. However, knockdown of c-FLIP led to an increase in RelA/p65 phosphorylation, indicating that c-FLIP inhibits NF-κB in ccRCC, which is consistent with reports showing an inhibitory role of c-FLIP on NF-kB in the context of death receptor signaling^25–27^. Therefore, the CD95L–CD95 system appears to activate NF-κB-dependent survival signaling in ccRCC cells, whereas c-FLIP blocks CD95L-induced apoptosis and dampens NF-κB activity. Nevertheless, c-FLIP allows NF-κB activation up to a level that is sufficient for expression of survival factors. Next, to c-FLIP-blocking apoptosis, additional NF-κB target genes might affect proliferation, angiogenesis, and other processes that support tumor progression^50,51^.

Although CD95 was originally regarded solely as a death receptor^52^, recent evidence suggested that it can have other, non-apoptotic functions^53^. For instance, it was shown that CD95 can actually promote tumor formation in mouse models of liver cancer and ovarian cancer and knockdown of CD95 in cancer cell lines impaired their proliferation^54^. In this regard, elevated CD95 expression was linked to later stages of RCC tumor progression, suggesting a beneficial effect of death receptor-mediated signaling for tumor cells^55–58^. Interestingly, we were not able to generate clearCa cells with a stable knockdown of CD95 (data not shown). At first, this latter observation is reminiscent of the recently described death induced by CD95 or CD95 ligand elimination (DICE)^59^. Indeed, there are certain similarities between DICE and the cell death we observe upon c-FLIP knockdown in ccRCC cells. For instance, DICE is associated with DNA fragmentation, vacuolization of the cells and caspase activation^59^. However, there are also clear differences. As such, DICE progresses independent of caspase activation, as it still occurs when caspases are blocked or in caspase-8-deficient cells^59^. Moreover, DICE was described as a necrotic form of cell death. In contrast, the cell death we observed upon c-FLIP knockdown in clearCa cells exhibited classical signs of apoptosis such as caspase activation, phosphatidylserine exposure, and formation of apoptotic bodies. Furthermore, c-FLIP knockdown-induced cell death of ccRCC cells was inhibited by the pan-caspase inhibitor QVD but not by the necroptosis inhibitor Nec-1. Our data are rather in line with a study on lung adenocarcinomas, which showed that targeting CD95, c-FLIP, or NF-κB signaling by RNA interference sensitized lung cancer cells towards EGFR tyrosine kinase inhibitors^60^. Thus, also other cancer cell types might use a CD95-c-FLIP-NF-κB circuit for promotion of survival and growth. Nevertheless, ccRCC cells appear to be special as depletion of c-FLIP or inhibition of CD95 signaling alone was sufficient to induce cell death in these cells.

Summarized, we demonstrated that c-FLIP mediates resistance to CD95L-induced apoptosis and that loss of c-FLIP leads to spontaneous apoptosis in RCC. In addition, loss of CD95 signaling also drives cells into spontaneous apoptotic cell death. We suggest a model in which paracrine CD95 stimulation leads to NF-κB activation to promote tumor growth in clearCa cells. The function of c-FLIP is to prevent CD95L-induced apoptosis. Although c-FLIP dampens NF-κB activation, it still allows a level of NF-κB activity that is sufficient to allow for expression of survival genes. Upon loss of this pathway, cells undergo spontaneous apoptosis owing to missing pro-survival signals. Our findings that c-FLIP and CD95 are important key regulators in survival of ccRCC cells might be used for new therapeutic approaches to overcome apoptosis resistance in RCC by downregulating their expression. This approach is independent of cell death-activating agents, minimizing side effects on normal tissue and might be also helpful for treatment for other tumor types.

## Materials and methods

### Reagents

Recombinant Flag-tagged CD95L was produced by transient transfection of HEK293T cells. The concentration of CD95L in the supernatant was determined by quantitative immunoblotting using purified Flag-FasL as a standard. CHX was purchased from Sigma Aldrich, QVD-oph was from MP Biomedicals and Nec-1 was from Enzo Life Sciences. The MEK1 inhibitor PD0325901 and the IKK2 inhibitor TPCA were purchased from Biozol/TargetMol.

### Cell culture and transient transfections

Immortalized ccRCC cell lines were generated from RCC tumor dissections and have been described previously^35,61,62^. RCC cell lines clearCa-2, 3, 4, and 6, and human embryonic kidney cells (HEK293T) were cultured in Dulbecco’s modified Eagle’s medium (DMEM high glucose; Invitrogen), supplemented with 10 % fetal calf serum (PAA Laboratories), 50 U/mL penicillin, and 50 µg/mL streptomycin. Transient transfection of HEK 293 T cells was done with HBS (0.28 m NaCl, 0.05 m HEPES, 1.5 mm Na_2_HPO_4_, pH 7.0) and 2.5 m CaCl_2_ according to standard protocols. Lentivirus-containing supernatant was harvested after 48 and 72 h post transfection.

### Lentiviral infection of cells

c-FLIP MISSION TRC shRNA constructs specific for all isoforms (Δc-FLIP_L/S_) or specific for c-FLIP_L_ (Δc-FLIP_L_) were purchased from Sigma. The generation of the c-FLIP_S_-specific shRNA construct (Δc-FLIP_S_) and the procedure of lentiviral transduction was previously described^34,63^. In brief, lentiviral vectors were co-transfected with the envelope vector pMD2.G (Addgene no. 12259) and the gag-pol expression plasmid pCMV_dR8.2dvpr (Addgene no. 8455) into HEK293T cells as described above. The supernatant was filtered through 0.45 µm polyvinylidene fluoride (PVDF) filters (GE Healthcare) and stored at 4 °C. For c-FLIP isoform-specific knockdown clearCa cells were transduced with 100 µL lentivirus-containing supernatant and 5 µg/mL polybrene (Sigma Aldrich). Cells were analyzed 3 days post transduction if not stated otherwise.

### Generation of a c-FLIP_RESIST_ re-expression plasmid

The puromycin resistance cassette of pLKO.1, containing c-FLIP_L/S_-specific shRNA (Sigma Aldrich) was exchanged by a mutated c-FLIP gene sequence (c-FLIP_RESIST_), avoiding the targeting by the c-FLIP_L/S_-specific shRNA to re-express functional c-FLIP_L_. The mutated c-FLIP_L_ gene was generated with the following primers: c-FLIPL_fwd: 5′-CGAGGATCCACCGGAGCTTACCATGTCTGCTGAAGTCATCC-3′; c-FLIPL_fwd2: 5′- CGAGGATCCACCGGAGCTTAC-3′; c-FLIPL_rev: 5′- GCTGGTACCTTATGTGTAGGAGAGGATAAG-3′; c-FLIP_mut_fwd: 5′- CCCTCACTTGGTCAGCGACTATAG-3′; c-FLIP_mut_rev: 5′- CTATAGTCGCTGACCAAGTGAGGG-3′. Gene mutation was performed by PCR with Phusion Flash II DNA polymerase (ThermoFisher Scientific). For cloning, the amplified c-FLIP_RESIST_ gene was cleaved with the restriction enzymes *Kpn*I and *Bam*HI (New England Biolabs).

### Immunoblotting

Cells were harvested with Trypsin-EDTA (ethylenediaminetetraacetic acid; Gibco® - Life technologies,) and lysed with TPNE buffer (1 % v/v Triton X-100 and 1 mm EDTA in phosphate-buffered saline (PBS) adjusted to 300 mm NaCl), supplemented with 1 mm phenylmethylsulfonyl fluoride, 1 µg/mL leupeptin, aprotinin, chymostatin, and pepstatin A. Protein concentrations were determined by Pierce BCA Protein Assay Kit (ThermoFisher Scientific) and up to 40 µg protein content were loaded on a 12% polyacrylamide gel and proteins separated by their molecular weight. After transfer onto a PVDF membrane (GE Healthcare), the membrane was blocked with 5% non-fat dry milk in TBS supplemented with 0.05 % Tween-20 (TBS-T) and incubated with primary antibodies overnight at 4 °C. After washing with TBS-T, the membrane was incubated with secondary antibodies, coupled with horseradish peroxidase for one hour at room temperature. After a second washing step, the membrane was developed by chemiluminescence (GE Healthcare). For reusing, the blots were stripped with Re-Blot Plus solution (Millipore). Primary antibodies for specific detection of proteins were: β-Actin (Ac-74, Sigma Aldrich), Bcl-x (Polyclonal, Transduction Laboratories), Caspase-8 (12F5, Dr. Klaus Schulze-Osthoff, Tübingen), Caspase-3 (Polyclonal, R&D Systems), CD95 (C-20, Santa Cruz), c-FLIP (NF6, Adipogen), Cleaved Caspase-3 (Asp175, 9661, Cell Signaling Technology), Cleaved Caspase-8 (18C8, Cell Signaling Technology), FADD (1F7, Upstate), FADD (TA332936, Origene), PARP (4C10–5, BD Biosciences), phospho-Erk1/2 (4370, Cell Signaling Technology), phospho-p65 (3033, Cell Signaling Technology), Tubulin (DM-1A, Sigma Aldrich), XIAP (48, BD Bioscience). Horseradish peroxidase-coupled secondary antibodies: anti-mouse IgG (sc-2055, Santa Cruz), anti-mouse IgG1 (1070–05, SouthernBiotech), anti-mouse IgG2a (1080–05, SouthernBiotech), anti-mouse IgG2b (1090–05, SouthernBiotech), anti-goat IgG (6160–05, SouthernBiotech), anti-rabbit IgG (4030–05, SouthernBiotech), anti-rat IgG (3050–05, SouthernBiotech).

### Quantitative real-time PCR

For detection of c-FLIP mRNA, cells were harvested and RNA isolated by RNeasy Plus mini Kit (Qiagen). After generation of cDNA with RevertAid RT Kit (ThermoFisher Scientific), real-time PCR was carried out with the 2× SYBR Green Kit (Roche) with c-FLIP-specific primers in a LightCycler 96 System (Roche). β-Actin was used as reference gene. Primers: c-FLIP_WT_fwd: 5′-AACCCTCACCTTGTTTCG-3′; c-FLIP_MUT_fwd: 5′-AACCCTCACTTGGTCAGC-3′; c-FLIP_rev: 5′-AACTCAACCACAAGGTCCA-3′; β-Actin_fwd: 5′-TGTTACCAACTGGGACGACA-3′; β-Actin_rev 5′-TCTCAGCTGTGGTGGTGAAG-3′.

### Flow cytometry

Cell surface staining was described previously^34^. For detection of dead cells, the DNA of untreated, CD95L-treated or lentiviral-transduced cells were stained with propidium iodide and measured in the PE channel. To assess apoptotic cells, the PE Active Caspase-3 Apoptosis Kit (BD Biosciences) was used, or cells were stained with AnnexinV (BD Biosciences) and 7AAD (Enzo Life Science) to discriminate between apoptotic and necrotic cells. For surface staining, specific antibodies against CD95 (2R2, Dr. K. Schulze-Osthoff, Tübingen, Germany), CD95L (5G51, Dr. K. Schulze-Osthoff, Tübingen, Germany), TNF-R1 (H398, Dr. H. Wajant, Würzburg, Germany), TRAIL (2E5, Enzo), TRAIL-R1 (DJR1, Biolegend) and TRAIL-R2 (DJR2–4, Biolegend) were used and detected with PE-coupled anti-mouse IgG-PE (115–116–146, Jackson Immuno Research Inc.).

### Fluorescence microscopy

Cells were seeded on microscopy glass coverslips and fixed with 3 % paraformaldehyde. After washing with PBS, the coverslips were incubated in primary antibody solution overnight at 4 °C. As primary antibodies anti-CD95 (2R2, Dr. K. Schulze-Osthoff, Tübingen, Germany) and anti-TNF-R1 (H398, Dr. H. Wajant, Würzburg, Germany) were used. After washing the coverslips with PBS, the coverslips were incubated in secondary antibody solution (anti-mouse IgG, A-11005, ThermoFisher Scientific) for1 hour at room temperature. The nuclei were stained with DAPI and pictures taken with an Eclipse Ti (Nikon instruments), supplied with an UltraViewVox Spinning Disc from PerkinElmer and analyzed with Volocity 3D Image (PerkinElmer).

### Resazurin assay

After washing the cells with PBS, a 10% solution of 0.2 mg/ml Resazurin sodium salt (Sigma) in PBS was added to fresh medium. After 3 h incubation in the dark at 37 °C, the fluorescence was measured at a Tecan infinite 200 reader (Ex/Em 540/590 nm).

### Statistics

Statistical analyses were performed with the software GraphPad Prism (GraphPad Software). Significances were calculated with one- or two-tailed nonparametric Mann–Whitney *U* test and Kruskal–Wallis one-way analysis of variance test.
